# Selective excitation enables encoding and measurement of multiple diffusion parameters in a single experiment

**DOI:** 10.5194/mr-2-835-2021

**Published:** 2021-11-19

**Authors:** Neil MacKinnon, Mehrdad Alinaghian, Pedro Silva, Thomas Gloge, Burkhard Luy, Mazin Jouda, Jan G. Korvink

**Affiliations:** 1 Institute of Microstructure Technology (IMT), Karlsruhe Institute of Technology, Hermann-von-Helmholtz-Platz 1, 76344 Eggenstein-Leopoldshafen, Germany; 2 DeepSpin GmbH, Kurfürstenstraße 56, 10785 Berlin, Germany; 3 Institute of Biological Interfaces (IBG-4), Karlsruhe Institute of Technology, Hermann-von-Helmholtz-Platz 1, 76344 Eggenstein-Leopoldshafen, Germany

## Abstract

Band selectivity to address specific resonances in a spectrum enables one to encode individual settings for diffusion experiments. In a single experiment, this could include different gradient strengths (enabling coverage of a larger range of diffusion constants), different diffusion delays, or different gradient directions (enabling anisotropic diffusion measurement). In this report, a selective variant of the bipolar pulsed gradient eddy current delay (BPP-LED) experiment, enabling selective encoding of three resonances, was implemented. As proof of principle, the diffusion encoding gradient amplitude was assigned a range dependent on the selected signal, thereby allowing the extraction of the diffusion coefficient for water and a tripeptide (Met-Ala-Ser) with optimal settings in a single experiment.

## 1 Introduction

There is little dispute over the importance of diffusion as a physical effect and its influence on many natural processes.

Diffusion poses a limiting factor in many industrial processes, such as the mixing of chemical reagents to achieve a specific product with sufficient yield. The interplay between intrinsic diffusion rates and operational time-dependent parameters needs to be properly understood and carefully considered in designing such manufacturing processes. Nuclear magnetic resonance (NMR) has proven to be uniquely capable to extract diffusion constants for a specific chemical substance and, in favourable cases, even mixtures, and also to study and understand the processes of diffusion at all length scales and within all compartments available to the molecule under consideration. Thus, NMR has facilitated experimental proof of Fick's laws and has facilitated precise measurements of anisotropic effects arising from spatially extended molecules in solution and subtracting the effects due to geometry, or ionic charges, and the like.

In magnetic resonance imaging, diffusion is currently the limiting spatial resolution factor for inductive NMR detection because excited spins in a molecule tend to diffuse away from their excitation site while waiting for pulse readout. Whereas this uncertainty cannot be removed completely, shorter pulse sequences have the effect of improving resolution by reducing the distance a spin ensemble can wander.

Spatial resolution is especially important in such areas as brain science and brain diagnostics, where anisotropic diffusion tensor imaging is the primary noninvasive means to discover the subcellular structure of a specific brain [Bibr bib1.bibx10]. Essentially, diffusing spin ensembles do not readily traverse cell walls and axons, so that the local structure of brain tissue renders an anisotropic response, with directional weighting being in favour of gliding along the cell walls. In this way, diffusion has helped to reveal brain connectivity patterns. But, also in studies of nanoporous materials, anisotropic diffusion parameters have been used to reveal local nanostructure.

Diffusion anisotropy measurements aim to reveal the 3-dimensional pattern of molecular movement. Mathematically, the movement at a spatial point has to be resolved in three orthogonal directions, which typically would require at least three motionally sensitive measurements, each in combination with a spatial gradient aligned with the specific direction of measurement. The most time-consuming part of the pulse sequence is the echo time and the recovery time. Spatial encoding will, additionally, require the three measurements for each spatial voxel, further slowing down the acquisition.

A degree of freedom available to spectroscopic diffusion measurements is the spectral dispersion, taking advantage of the individual resonances as a means of encoding additional information within a single experiment. This is particularly interesting for molecular mixtures whose components vary in the physical dimensions and, thus, their diffusion coefficient, or for a tracer molecule in an anisotropic environment, where diffusion is dependent on direction. Instead of performing multiple diffusion measurements, each optimized for a particular regime, a frequency bandwidth could be selectively encoded with appropriate diffusion parameters independent of another, different bandwidth, which is itself encoded with differing diffusion parameters. This could be extended down to encoding individual resonances in a spectrum with diffusion parameters appropriate for extracting diffusive properties with high precision.

There are two additional benefits to integrating selective elements to the experiment: first is the elimination of dominating, uninteresting signals (typically solvent) to improve sensitivity to minor components. This has been demonstrated for protein diffusion in water using a selective version of the stimulated echo experiment [Bibr bib1.bibx28] and a selective version of the spin echo experiment for measuring minor components in a mixture [Bibr bib1.bibx13]. The second benefit, noted by [Bibr bib1.bibx28] and [Bibr bib1.bibx13], is to reduce spectral congestion and, thereby, improve diffusion ordered spectroscopy (DOSY) data analysis, an active field of research in extracting diffusion coefficients from overlapping signals [Bibr bib1.bibx1].

In this report, we have extended the stimulated echo experiment using bipolar gradients and longitudinal eddy current compensation BPP-LED; to enable selective diffusion encoding. As proof of concept, we demonstrate the ability to selectively address up to three individual resonances (each with a bandwidth of 60 Hz), each encoded with independent diffusion parameters.

In this paper we are specifically paying tribute to Geoffrey Bodenhausen, a pioneer of NMR spectroscopy, on the occasion of his 70th birthday. Our chief source of inspiration comes from one of his earliest papers, published in 1976 [Bibr bib1.bibx3] while he was at Oxford, which explores selective excitation, and many other of his papers that have explored the measurement of diffusion by various ingenious means.

## 2 Materials and methods

### 2.1 Materials

Deuterium oxide ( $D_{2}O$ ) and the tripeptide Met-Ala-Ser (MAS) were purchased from Sigma Aldrich and used as received. All $H_{2}O$ was of MQ quality. An NMR sample in a 5 mm NMR tube was prepared by dissolving 10 mg MAS in 500 µL of 90:10 $D_{2}O$ : $H_{2}O$ .

### 2.2 NMR spectroscopy

NMR experiments were performed using an AVANCE III 500 MHz wide bore NMR spectrometer (Bruker BioSpin GmbH, Ettlingen, Germany) using a Micro5 micro-imaging probe equipped with a 5 mm NMR detector. The sample temperature was controlled using cooling water flowing through the imaging gradient sleeve, and it was maintained at 20 ${}^{\circ }$ C.

Non-selective diffusion experiments were performed using the bipolar gradient longitudinal eddy current compensated experiment BPP-LED; Bruker pulse program ledbpgp2s1d. The diffusion-encoding gradient pulse was the smoothed square (SMSQ10.100), with 2 ms length, 200 µs gradient recovery time, and gradient strength was varied using the parameter optimization (popt) function in TopSpin 3.6.3 (Bruker BioSpin GmbH, Ettlingen, Germany) from 1 % to 95 % of the maximum gradient strength through 16 experiments, and the diffusion time was 250 ms.

**Figure 1 Ch1.F1:**
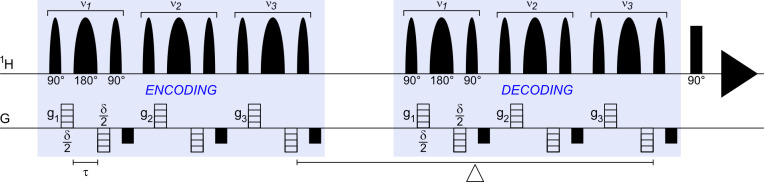
The selective BPP-LED (bipolar pulse paired longitudinal eddy current delay) diffusion experiment, i.e. Sel-BPP-LED. In this work, three spectral regions with centre frequency $\nu_{n}$ were encoded. For each selective spectral region, $g_{n}$ were defined separately for $\nu_{1}$ (water) and $\nu_{2,3}$ (MAS peptide; Met and Ala methyl signals). Despite a hard 90 ${}^{\circ }$ read pulse using the same phase cycle as the BPP-LED sequence, the resulting spectrum consists of only the selected spectral regions (Fig. [Fig Ch1.F2]). While not demonstrated in this work, diffusion encoding for each selected frequency could be uniquely defined beyond using $g_{n}$ by including $\delta_{n}$ and inserting additional delays between the $\nu_{n}$ decoding segments to vary $\Delta_{n}$ and the direction of g (where hardware permits). All parameters used this work are described in Sect. 2.

Selective diffusion experiments were performed using a modified version of the non-selective experiment (Sel-BPP-LED); 90 ${}^{\circ }$ hard pulses were replaced by sinc-shaped pulses, and 180 ${}^{\circ }$ refocusing pulses were replaced by Gaussian refocusing pulses (Sinc1.1000 and Gaus1_180r.1000 from the Bruker shaped pulse library; Fig. [Fig Ch1.F1]). The selective pulse length and power were calculated using the Shape Tool (TopSpin 3.6.3), using a selective bandwidth of 60 Hz, resulting in excitation pulses of 26.8 ms and refocusing pulses of 14.7 ms. The diffusion time was Δ=250 ms, with the timing in the pulse program modified by taking into account the additional selective blocks in the pulse sequence. In total, three signals were chosen for the selective diffusion experiment, namely the water signal (4.8 ppm – parts per million) and, from the MAS peptide, the methyl signal of methionine (2.1 ppm) and the methyl signal of alanine (1.45 ppm). As in the non-selective experiment, the popt function was used to vary the maximum gradient amplitude over 16 experiments. The simultaneous optimization feature of the popt function was used to control the three gradient amplitudes, $g_{n}$ , individually (Fig. [Fig Ch1.F1]).

All NMR experiments were performed using radiofrequency (RF) pulses calibrated on the day of measurement. Each measurement consisted of eight scans, with each scan containing 32 K data points over a spectral width of 20 ppm. The free induction decays (FIDs) were zero filled by a factor of 2 and multiplied by an exponential function equivalent to 0.3 Hz line width prior to Fourier transformation. The series of 16 spectra from each diffusion measurement was extracted using an in-house MATLAB script (MATLAB, 2020b; MathWorks, Natick, MA, USA). The MATLAB fit function was used to extract the diffusion coefficients. We note that the DOSY functionality available in TopSpin was neither used in setting up the experiments nor in the data analyses.

Diffusion coefficients were extracted by fitting the intensity versus applied gradient strength using the following equation [Bibr bib1.bibx27]:

1 $$I=\mathrm{exp}\left(-\gamma^{2}G^{2}\delta^{2}D(\Delta -\delta /3-\tau /2)\right),$$

where I is the normalized NMR signal intensity, γ is the ${}^{1}$ H gyromagnetic ratio, δ is the diffusion gradient pulse length, Δ is the diffusion time, τ is the time between the bipolar gradient pulses, and G is the applied gradient strength. It is noted that the value of τ becomes significant in the selective experiment (14.9 ms in this work), and that the equation would need to be specified separately for each selective frequency in case different selective pulse bandwidths, and, therefore, different refocusing pulse lengths, are to be used. There was no correction applied for using the smoothed square gradient shape which, it should be noted, deviates slightly from an ideal rectangular pulse [Bibr bib1.bibx24]. The diffusion of water in the $D_{2}O$ : $H_{2}O$ mixture was used to calibrate an apparent gradient strength assuming (i) a diffusion coefficient $D=2.02\times 10^{-9}$ m ${}^{2}$ /s taken for pure $H_{2}O$ ; and (ii) rectangular gradients. The apparent gradient strength was determined to be 260 G cm ${}^{-1}$ .

### 2.3 Results and discussion

A series of experimental NMR spectra measured, using both BPP-LED and Sel-BPP-LED, is presented in Fig. [Fig Ch1.F2], with a summary of the details provided in Table [Table Ch1.T1]. Comparing the signal-to-noise ratio (SNR) when using the BPP-LED vs. Sel-BPP-LED experiments, the water signal decreased by 33 %, while the MAS methyl signals of methionine and alanine were essentially unchanged ( - 4 % and + 2 %, respectively). The Sel-BPP-LED experiment yielded intensity decay curves faithfully reproducing the non-selective experiment (Fig. [Fig Ch1.F3]a). A slight variation in the extracted diffusion coefficients was observed (3.3 %, 3.9 %, and - 1.5 % relative deviation for $D_{\mathrm{water}}$ , $D_{\text{MAS,Met}}$ and $D_{\text{MAS,Ala}}$ ). The likely source for the deviations is selective RF pulse imperfections, which could be compensated by using selective pulse shapes with better performance, for example Gaussian pulse cascades Q 3 and Q 5 [Bibr bib1.bibx7] or optimal control derived selective pulses [Bibr bib1.bibx20]. In the case of all three signals being exposed to 1 %–95 % of gradient maximum, the extracted water diffusion coefficient was $(19.2\pm 0.2)\times 10^{-10}$ m ${}^{2}$ s ${}^{-1}$ , a value with larger fit error (1.1 % vs. 0.37 %) and slightly larger deviation relative to the non-selective experiment (4.0 % vs. 3.3 %) compared to using a gradient strength spanning 1 %–60 %. The extracted values for the MAS Met and Ala methyl signals were essentially unchanged ( $(4.16\pm 0.01)\times 10^{-10}$ m ${}^{2}$ s ${}^{-1}$ and $(4.20\pm 0.01)\times 10^{-10}$ m ${}^{2}$ s ${}^{-1}$ ).

**Table 1 Ch1.T1:** Non-selective (BPP-LED) and selective (Sel-BPP-LED) diffusion experiments are compared. Water (4.8 ppm), the methionine methyl signal of MAS (2.0 ppm), and the alanine methyl signal of MAS (1.4 ppm) were used for these analyses. SNR was calculated using the first increment in the diffusion series. Diffusion coefficients were extracted from the diffusion plots using Eq. ([Disp-formula Ch1.E1]). The diffusion time was the same for all experiments ( Δ= 250 ms). A single Sel-BPP-LED experiment was used, with the water and MAS signals exposed to gradient strengths ranging from 1 %–60 % and 1 %–95 % of maximum, respectively. Reported SNR values are the mean ± standard deviation of triplicate measurements.

Signal	Selective	SNR	Diffusion coefficient
	(yes/no)	$\left(\times 10^{3}\right)$	( $\times 10^{-10}$ m ${}^{2}$ s ${}^{-1}$ )
Water	N	2700±700	20.0±0.6
Water	Y	1800±130	19.3±0.07
Met	N	100±30	4.33±0.03
Met	Y	102±8	4.17±0.01
Ala	N	90±20	4.14±0.02
Ala	Y	86±6	4.20±0.01

The increased degree of experimental freedom enabled by Sel-BPP-LED is demonstrated in Figs. [Fig Ch1.F2]b–c and [Fig Ch1.F3]b. Figure [Fig Ch1.F2]b reveals that the water signal fully decays well before the MAS when using the same gradient amplitude across all signals. Adjusting the gradient amplitude range for the water signal to apply 1 %–60 % of maximum strength was found to be sufficient to reach full decay at the end of the experiment. In the same experiment, the MAS signals were simultaneously exposed to gradient amplitudes ranging from 1 %–95 % of maximum strength, which was appropriate to sample the intensity decay curve for the larger molecule. As a result, the Sel-BPP-LED experiment enables parallel diffusion measurements of molecules of vastly different diffusive properties by selectively encoding the appropriate diffusion experimental parameter (i.e. gradient amplitude in this case) into a well-resolved signal.

Selective variants of numerous NMR experiments have been realized since the idea of converting multi-dimensional to 1D experiments using selective pulses was reported [Bibr bib1.bibx14]. Such experiments are often exploited to simplify otherwise complex spectra [Bibr bib1.bibx15], to suppress strong signals that dominate the dynamic range [Bibr bib1.bibx6], or to selectively drive desired coherence pathways [Bibr bib1.bibx11]. Diffusion experiments have also benefited from the addition of selectivity to reduce the influence of strong solvent signals in biomolecular samples [Bibr bib1.bibx23], to focus the diffusion experiment onto select molecules in a complex mixture [Bibr bib1.bibx17], or to use the diffusion dimension as an access point to further selective-based NMR experiments e.g. selective TOCSY – Total Correlation Spectroscopy; and supplemented with relaxation encoding; Poggetto et al., 2017.

The selective experiment described in this report offers the same advantages in terms of signal suppression and reducing complexity. A key difference compared to previous selective diffusion reports is the encoding of information into individual resonances. This is in contrast to the method described by [Bibr bib1.bibx13], who demonstrated diffusion measurements by the selective excitation of four resonances. Since every resonance was exposed to the same experiment parameters, all four resonances could be simultaneously excited resulting in a net gain in time per scan compared to the experiment described here. [Bibr bib1.bibx28] demonstrated a selective diffusion experiment using selective pulses of larger bandwidth ( ∼ 2 kHz) and, therefore, also gained in experimental time per scan. The benefit of the Sel-BPP-LED experiment (in fact, we are not limited to BPP-LED; any stimulated echo based-diffusion experiment should be compatible with the concept) is the ability to parallelize measurements, thus accessing diffusion information from multiple sample species in a single experiment.

**Figure 2 Ch1.F2:**
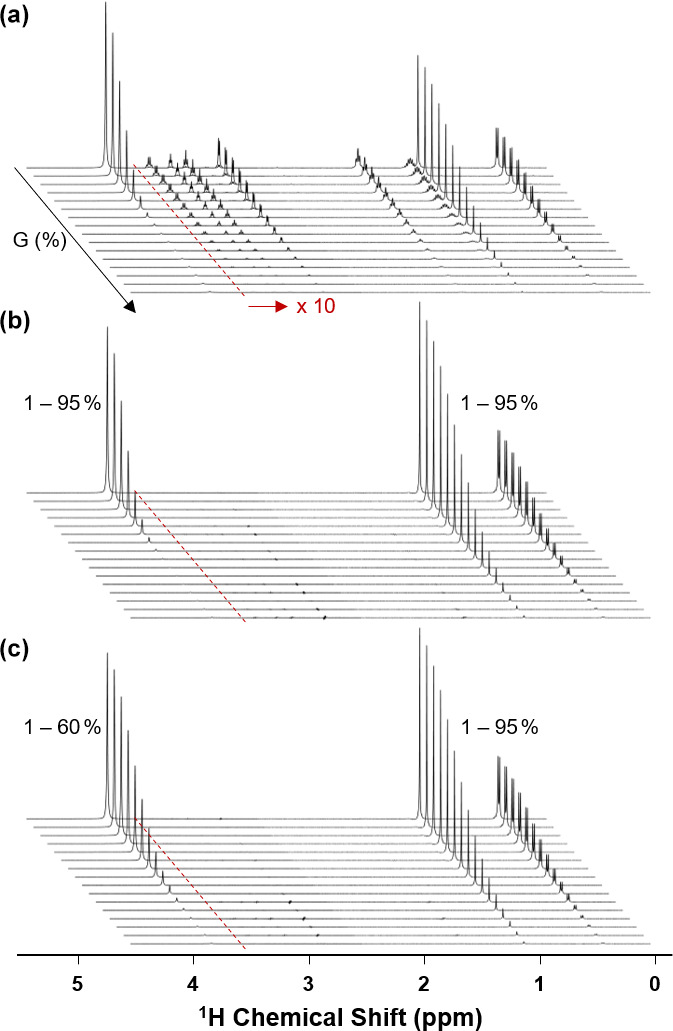
Exemplary ${}^{1}$ H NMR spectra of 20 mg mL ${}^{-1}$ Met-Ala-Ser (MAS) tripeptide non-selective **(a)** and selective **(b, c)** diffusion experiments. The resonances used for selective encoding were water (4.8 ppm), the Met methyl group of MAS (2.1 ppm), and the Ala methyl group of MAS (1.5 ppm). As demonstrated in panel **(c)**, using selective encoding, the gradient amplitude experienced by the water signal could be controlled independent of that experienced by the MAS signals (1 %–60 % vs. 1 %–95 %). In all plots, the intensity of the region from 1–4.5 ppm was increased by a factor of 10 for clarity (highlighted in panel **a**).

**Figure 3 Ch1.F3:**
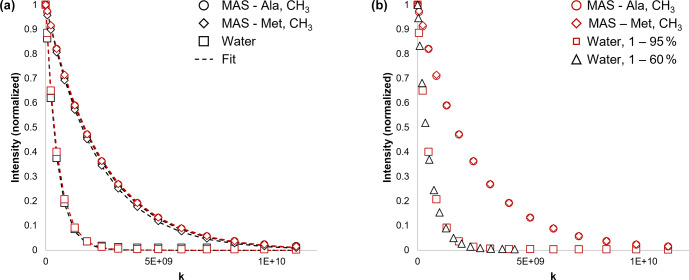
**(a)** Comparison of signal intensity versus the diffusion experiment parameter $k=\gamma^{2}G^{2}\delta^{2}(\Delta -(\delta /3)-(\tau /2))$ for the alanine and methionine methyl signal of Met-Ala-Ser (MAS; circles and diamonds) and the water signal (squares). Black symbols indicate that the experiment was non-selective; red symbols indicate that selective excitation was used. The dashed lines are the fits to the data using Eq. ([Disp-formula Ch1.E1]). The adjusted $R^{2}$ values were all > 0.999. The diffusion time was Δ=250 ms. **(b)** Comparison of signal intensity versus $k=\gamma^{2}G^{2}\delta^{2}(\Delta -(\delta /3)-(\tau /2))$ for the water (squares and diamonds) and MAS Ala and Met methyl (circles and diamonds) signals using Sel-BPP-LED. Using the selective experiment, the applied gradient strength experienced by the water signal could be varied independently of the applied gradient strength experienced by MAS. Diamonds show that the applied gradient strength varied from 1 %–60 %. Using the selective excitation experiment, MAS signals were always exposed to an applied gradient varying from 1 %–95 %. The experiments were performed in triplicate, and the mean values are plotted. The diffusion time was Δ=250 ms.

The ability to accelerate measurements via parallelization will be important in many applications. For example, considerable advantages for increased measurement time efficiency for DTI (diffusion tensor imaging) applications are anticipated, since the 3D diffusion tensor (with up to six components) needs to be determined on a voxel-by-voxel basis. In this case, the ability to encode up to six scalar components in a single k -space acquisition, i.e. in one shot and subsequent FID, would be a tremendous advantage. For technical systems, this could be achieved by using fluids of designed composition, such that at least six well-resolved signals are available. For clinical DTI, the measurement will be reliant on identifying molecules that are sufficiently abundant and a chemical shift resolution at typical MRI scanner field strengths e.g. at 3 T, candidates could include lactate, N-acetyl amino acid derivatives, choline derivatives, or creatine derivatives;; however, even if all six tensor components cannot be encoded, there will still be an measurement acceleration when more than one component can be encoded. An additional example is complex samples containing both slow- and fast-diffusing species where restricted diffusion is present. Using a selective diffusion experiment, the optimal parameters can be simultaneously used for the fast-diffusing species and, in the example presented in Fig. [Fig Ch1.F4], the restricted slow-diffusing species.

**Figure 4 Ch1.F4:**
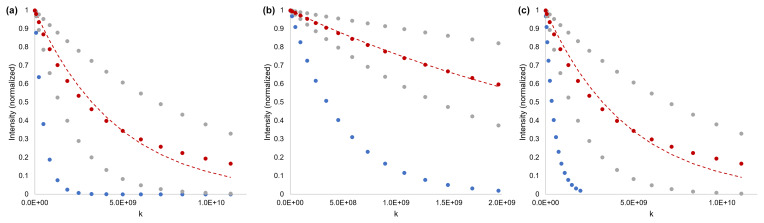
Simulated data to demonstrate the distinction between a fast-diffusing mixture component and a slow-diffusing mixture component experiencing restriction. **(a)** Gradient strength sufficient to observe multicomponent decay (failure of single exponential fit – red), but not optimized for the fast component (blue). **(b)** Gradient strength optimized for fast-diffusing species (blue) but not sufficient to identify multi-component diffusion (red). **(c)** Single experiment and gradient strength optimized independently for fast- and multicomponent species. Dashed lines are fits to the diffusion equation (Eq. [Disp-formula Ch1.E1]), assuming a single exponential decay. The red dots are the sum (50 : 50) of two single exponential decays (grey). Simulated $D=2\times 10^{-9}$ m ${}^{2}$ s ${}^{-1}$ (blue), $5\times 10^{-10}$ m ${}^{2}$ s ${}^{-1}$ , and $1\times 10^{-10}$ m ${}^{2}$ s ${}^{-1}$ (grey), with G=260 .8 G cm ${}^{-1}$ , δ=2 ms, Δ=250 ms, and τ=0 .21 ms.

The limit to the number of selective units, n , in the selective diffusion experiment is governed by (i) the desired bandwidth to be selectively encoded, (ii) the desired diffusion time Δ , and, related, (iii) the $T_{1}$ relaxation of the selected signals. For the worst-case scenario, selectivity with small bandwidth of multiple resonances with short $T_{1}$ will not be possible since the resulting diffusion time would be too long, and signal intensity would be lost to relaxation. In the experiment demonstrated here, a selective bandwidth of 60 Hz was used exclusively, requiring selective pulse lengths of 26.8 ms and 14.7 ms for excitation and refocusing. This limited the diffusion times Δ>3×68.3 ms for the three signals selectively encoded or, more generally, $\Delta >n\times (2\times t_{90,\mathrm{sel}}+t_{180,\mathrm{sel}}+\delta )$ . Shorter diffusion times can be accessed by selectively encoding spectral regions instead of individual resonances, thereby reducing the required length of the selective pulse (in the case of J-coupled signals within the excited bandwidth, there will be minor signal loss to anti-phase coherence, which will be destroyed by the gradient applied after magnetization storage). This has been demonstrated for protein and peptide diffusion measurements by selectively exciting bandwidths of the order of 2 kHz with the benefit of relatively short selective pulse lengths of the order of 2.5 ms [Bibr bib1.bibx28]. Encoding more than one resonance with the same diffusion parameters would also be possible using pulses with multiband selectivity, which would effectively reduce n and also enable access to shorter diffusion times.

## 3 Conclusions

Taking advantage of individual resonances as a means to encode additional, unique information into a single experiment is a means to achieve measurement parallelization. For each unique encoding n , experimental time is accelerated by a factor n . In this work, a Sel-BPP-LED diffusion experiment demonstrated this concept, with n=2 in this case, since two signals belonged to the same molecule and were encoded with the same gradient direction. While demonstrated for the BPP-LED experiment, the concept is general and can be applied to any diffusion pulse sequence implementing the stimulated echo. Selective encoding enables the simultaneous measurement of diffusion coefficients of sample mixture components experiencing different transport properties dictated by molecular size or selective restriction via matrix interactions. This principle could be extended to anisotropic diffusion by encoding gradient directions into different resonances. To improve the experiment, selective RF pulses with better performance should be explored to avoid systematic deviations in the extracted diffusion coefficients.

## Data Availability

The Sel-BPP-LED pulse program, tested using TopSpin 3.6.3, and the NMR data are available at https://doi.org/10.5281/zenodo.5105713 (MacKinnon et al., 2021).
